# Nanoemulsion and nanoencapsulation of a hydroethanolic extract of Nettle (*Urtica dioica*) and Wormwood (*Artemisia absinthium*): comparison of antibacterial and anticancer activity

**DOI:** 10.3389/fchem.2024.1266573

**Published:** 2024-01-16

**Authors:** Zeinab Rahmani, Merat Karimi, Iman Saffari, Hamed Mirzaei, Majid Nejati, Reza Sharafati Chaleshtori

**Affiliations:** ^1^ Department of Laser and Photonics, Faculty of Physics, University of Kashan, Kashan, Iran; ^2^ Institute of Nanoscience and Nanotechnology, University of Kashan, Kashan, Iran; ^3^ Department of Food Hygiene and Quality Control, Faculty of Veterinary Medicine, Science and Research Branch, Islamic Azad University, Tehran, Iran; ^4^ Research Center for Biochemistry and Nutrition in Metabolic Diseases, Kashan University of Medical Sciences, Kashan, Iran; ^5^ Anatomical Sciences Research Center, Institute for Basic Sciences, Kashan University of Medical Sciences, Kashan, Iran

**Keywords:** *Urtica dioica*, *Artemisia absinthium*, antibacterial activity, anticancer activity, hydroethanolic extract, nanoemulsion, nanoencapsulation

## Abstract

**Introduction:** Nanoemulsion and nanoencapsulation are attractive novel methods that can be used for incorporating active plant extracts in food preparations and pharmaceutical formulations. In the current study, we aimed to investigate the anticancer and antibacterial effects of hydroethanolic extracts of Nettle (NE), Wormwood (WE), and the combination of the two plants (CNWE), as well as their nanoemulsion forms (NN, NW, CNNW) and nanoencapsulation forms (CN, CW, and CCNW).

**Methods:** The morphology and structure of the nanoemulsion and nanoencapsulation preparations were assessed utilizing dynamic light scattering (DLS) along with transmission electron microscopy (TEM). The antibacterial activity of the prepared formulations were assessed by determining minimum inhibitory concentration (MIC), zone of inhibition diameter, minimum bactericidal concentration (MBC), along with biofilm growth inhibition against Salmonaella typhimurium and Klebsiella. pneumoniae. The anticancer activity was evaluated via a MTT assay in the colon cancer cell line (HCT116).

**Results:** The nanoemulsion and nanoencapsulation particle size varied between 10 and 50 nm and 60 and 110 nm, respectively. The MIC values were between 11.25 and 95 µg/mL along with MBC values between 11.25 and 190 µg/mL. The highest inhibition of biofilm formation was observed with CCNW against K. pneumoniae (∼78.5%) and S. typhimurium (∼73%). In descending order, the inhibition of biofilm formation was CCNW > CW > CN > CNNW > NN > NW > CNWE > NE > WE against the tested bacteria. The IC50 values for NE, WE, CNWE, NN, NW, CNNW, CN, CW, and CCNW were determined as 250, 170, 560, 380, 312, 370, 250, 420, and 700 µg/mL, respectively. Exposure to a high concentration of NW resulted in a significantly lower HCT116 viability compared to other groups. Taken together, CNNW, and CCNW showed the highest antibacterial and anticancer activitiy.

**Discussion:** Nanoemulsion and nanoencapsulation were effective ways to increase the antibacterial and anticancer activity of the extracts and could be used in the food and pharmaceutical industries.

## Introduction

There is a long history of using plants to treat diseases, and many traditional medical therapies have their roots in the use of medicinal plants. Today, medicinal plants are attractive for many researchers due to their low side effects, variety of effective ingredients, strong anti-inflammatory and antioxidant activity, and their ability to prevent tumor growth (Chaleshtori et al., 2015; Wu et al., 2023). In this regard, in recent decades, plants have become globally significant sources of new anticancer drugs and antibiotics (Kaur et al., 2023; Shehabeldine et al., 2023).

Nettle (*Urtica dioica*) is a widespread perennial herbaceous flowering plant found across the globe (Bhusal et al., 2022). This plant, belonging to the Urticaceae family, is a valuable source of minerals, amino acids, carotenoids, vitamins, flavonoids, tannins, sterols, and polysaccharides. It is important as a medicinal plant and is often recommended for inclusion in the human diet (Bhusal et al., 2022; Dağlıoğlu et al., 2023a). Numerous reports have shown the beneficial effects of Nettle (seeds and leaves) in treating various diseases, including diabetes, eczema, hepatitis, anemia, rheumatism, and prostate cancer. Additionally, the plant leaves have been used for treating high blood pressure as well as arthritis (Bhusal et al., 2022; Teixeira et al., 2023).

Wormwood, also known as *Artemisia absinthium*, belongs to Asteraceae family (Sohail et al., 2023). This plant can easily grow in various climate conditions in regions of Iran, including around Tehran (Damavand), Azerbaijan, Khorasan, and Gilan. It is cultivated and used in traditional Iranian medicine and around the world to treat liver failure, sore throat, ear infection, constipation, chronic diarrhea, wound healing, chronic fever, and can also serve as an antidote to insect bites (Mehrabani et al., 2016; Akram et al., 2023). Alpha and beta thujone are monoterpenes, which are the main components of the Wormwood plant, but are considered to be poisonous. Many pharmacological studies have reported that thujone is toxic, despite its use as a pain reliever and anthelmintic. High consumption of thujone can lead to nervous disorders, and it has been shown to exert its toxic effects via cannabinoid receptors (Pal and Dey, 2023).

Nanoemulsions are attractive formulations due to the small size of the droplets, long-term physical stability (no observable aggregation, bi-phase formation or precipitation) and transparency. They require less surfactant, promote higher absorption of antimicrobial compounds by microorganisms, leading to reduced consumption, which is advantageous. The production of nanoemulsions for microencapsulation and controlling the release of beneficial compounds, such as various drugs, dyes, essences, or vitamins, is an example of the ways nanotechnology is being used in the food industry (Naseema et al., 2021).

The technique of encapsulating aromatic compounds aims to protect them against light, heat, and oxygen, while reducing their volatility. This technique is used in the industry for creating capsules that allow controlled release in the digestive system at a specific time, speed, and location. Nanocapsules can also offer protection against other ingredients in food (English et al., 2023).

An earlier investigation revealed that the antibacterial activity of Nettle is more effective against Gram-negative bacteria like *Pseudomonas fragi* and *Campylobacter jejuni* strains, compared to other bacteria like *Escherichia coli*, *Shewanella putrefaciens*, *Listeria innocua*, etc. (Elez Garofulić, 2021). Another investigation revealed that a hot methanolic extract of *A. absinthium* leaves (HMEL) could actively inhibit the proliferation of breast cancer cells MCF-7, with an IC_50_ value of 80.96 ± 3.94 μg/mL. HMEL showed a moderate spectrum of antibacterial activity against both Gram-positive and Gram-negative bacteria (Sultan, Zuwaiel et al., 2020).

Nevertheless, so far no studies have been reported on the use of nanoemulsion and nanoencapsulation of Nettle and Wormwood to provide anticancer and antimicrobial activity. Therefore, we aimed to investigate the anticancer and antibacterial activity of NE, WE, and the CNWE hydroethanolic extracts, as well as their nanoemulsion forms (NN, NW, CNNW) and nanoencapsulation forms (CN, CW, and CCNW).

## Materials and methods

### Plant material and chemicals

The Nettle and Wormwood plants were obtained from an herbal market in Kashan, Iran. Ethanol, Tween 20, Tween 80, tetrazolium bromide solution (MTT), polyethylene glycol, phosphotungstic acid, crystal violet, L-glutamine, starch and chitosan were obtained from Sigma Aldrich Chemical Co. (St. Louis, MO, United States). Dimethyl sulfoxide (DMSO) was provided by Merck, Darmstadt, Germany. Nutrient agar (NA), Mueller Hinton agar (MHA) and broth (MHB) as well as brain heart infusion (BHI) were purchased from HiMedia, Mumbai, India. Fetal bovine serum (FBS) and penicillin/streptomycin were obtained from Hyclone, Logan, UT, United States. Gentamicin (10 µg) was purchased from Padtan Teb Co., Iran. Colon cancer cell line (HCT 116) was purchased from the Pasteur Institute of Iran. *S. enterica* subsp. *enterica* serovar *typhimurium* (ATCC 14028) and *K. pneumoniae* (ATCC 10031) were obtained from the Department of Clinical Medical Microbiology at Kashan University of Medical Sciences.

### Extraction using hydroethanolic solvent

The seeds, bark, and leaves were carefully cleaned to remove any dust, followed by drying the leaves under shade for 2 weeks. The dried leaves were then powdered using a mixer grinder, and the powder was macerated with 80% ethanol to produce the extract (Rafieian, 2010; Bonilla and Sobral, 2016). Briefly, 400 mL of 80% ethanol was combined with 100 g of each powder and allowed to settle at ambient temperature (22°C ± 2°C) for 8 h. The resulting Nettle and Wormwood extracts (NE and WE) were collected, filtered, concentrated, and stored in the refrigerator.

### Nanoemulsion and nanoencapsulation preparations of Nettle (NE and CN) and Wormwood (WE and CW)

The nanoemulsion and nanoencapsulation formulations were prepared using Nettle and Wormwood extracts, with Tween 20 and Tween 80 as emulsifiers, polyethylene glycol as an auxiliary solvent, and distilled water. To achieve a stable nanoemulsion, the ratio of emulsifier to extract was varied from 0.1 to 2. The results showed that a ratio of 0.6 produced the most stable emulsion. The preparation of all nanoemulsions was carried out using an ultrasonic wave generator probe (Heshler, Germany) for a duration of 30 min. Chitosan and modified starch (nanostarch) were dissolved in 1% (v/v) acetic acid overnight on a magnetic stirrer (Pouya Electric, Iran) at room temperature, at a ratio of 8.5:1.5% (w/v). The previously created nanoemulsion was added dropwise to this solution to create a nanoencapsulation, which was utilized in the subsequent analysis.

### Particle size measurement

Transmission electron microscopy (TEM) analysis was conducted with a JEM-100CXII microscope from Hitachi Co. Ltd., Japan (Souza et al., 2016). To examine the structure of the nanoemulsion and nanoencapsulation, a droplet of each sample was deposited onto a carbon-coated copper wire and subsequently stained with a 2 wt% solution of phosphotungstic acid for 2 min. The stained droplets were allowed to air-dry for 10 min and subsequently examined with TEM at an acceleration voltage of 200 kV. The dynamic light scattering (DLS) method using a Zetasizer Nano ZS, model ZEN3500 from Malvern Instruments, United Kingdom, was employed for particle size analysis of the nanoemulsion and nanoencapsulation, (Souza et al., 2016). Each sample underwent the following preparation and analysis. A 100-fold dilution was prepared by adding 10 µL of sample to 990 µL of deionized water. The resulting mixture was then transferred to a disposable plastic cuvette for analysis. The analysis was conducted in triplicate at 25°C.

### Analysis of antimicrobial activity

Two Gram-negative bacteria were employed in this investigation: *S. typhimurium* (ATCC 14028) and *K. pneumoniae* (ATCC 10031). Initially, each bacterium was grown in NA for 18 h at 37°C. Next, an inoculum was prepared by adjusting the concentration to 0.5 MacFarland units in sterile saline (∼1 × 10^8^ CFU/mL) (Sharafati-Chaleshtori et al., 2010; Dinkov et al., 2016).

The MIC and MBC were determined by the microdilution method (Ali et al., 2021). A 10% (v/v) DMSO solution diluted in MHB was prepared as the growth media. In each row of a 96-well plate, the final two wells were designated as growth control and sterility control, respectively. Into each well, 5 µL of 0.5 McFarland suspension and 95 µL of MHB, were added. Next 100 µL of serial 2-fold dilutions of NE, WE, CNWE, NN, NW, CNNW, CN, CW, and CCNW were transferred to each well. The 96-well plates were placed in a shaking incubator (Noor Sanat Tajhiz Ferdows, Iran) and incubated for 20 h at 37°C. After that, the wells were checked for turbidity to measure bacterial growth. The plate containing the lowest concentration of NE, WE, CNWE, NN, NW, CNNW, CN, CW, and CCNW that resulted in no turbidity (indicating bacterial growth inhibition) was taken as the MIC. Additionally, the MBC was taken as the lowest concentration at which the MHA showed no discernible growth.

The disc diffusion test was conducted following the guidelines outlined in the CLSI M02-A11 document (CLSI, 2015). Briefly, a prepared inoculum of the mentioned bacteria (∼1 × 10^8^ CFU/mL) was spread onto solid MHA plates. Sterile Whatman paper N.1 discs with a diameter of 6 mm were used to add various concentrations of NE, WE, CNWE, NN, NW, CNNW, CN, CW, and CCNW, which were diluted in 10% (w/v) DMSO and incubated for 24-h at 37°C. A millimeter-scale ruler was used for measuring the width of the inhibition zones (IZ). For this technique, DMSO 10% and gentamicin (10 µg) were used as negative and positive controls, respectively. The equation below was used to calculate the IZ diameter (Muchtaromah et al., 2020):

$$Inhibitory\;zone\;diameter\;\left(mm\right)=Clear\;zone\;diameter-Paper\;disc\;diameter$$

### Antibiofilm activity

The bacterial biofilm formation capacity, as well as the anti-biofilm activity of NE, WE, CNWE, NN, NW, CNNW, CN, CW, and CCNW, were evaluated using a microtiter plate biofilm formation assay (Hassanshahian et al., 2020). To begin with, a bacterial suspension was prepared at a concentration of 10^6^ CFU/mL. This suspension was used to inoculate BHI media at sub-lethal concentrations (0.5, 1, and 2 MICs) of the above preparations. The plates were then incubated for a 24-h at 37°C without agitation, allowing the cells to adhere to each well surface. After incubation, the cells were removed by flipping the plate over and emptying out the contents. Subsequently, the supernatant was carefully aspirated from each well, leaving the intact biofilm behind. The remaining biofilm in each well was stained with 1% crystal violet solution. The wells were twice rinsed with 250 µL of sterile water to remove any remaining crystal violet after 30-min incubation at 37°C. The plates were air-dried at 37°C for 1 h. To dissolve the stained biofilm, 200 µL of ethanol (95%) was added, and the optical density (OD) was measured at 570 nm (OD 570 sample). To minimize background effects, stained wells with BHI medium (without any bacterial cells or samples) were employed as a negative control. Similarly, wells containing only bacterial cells (without any samples) served as a positive control. The biofilm inhibition percentage for each antibacterial agent was determined using the following equation:

$$\%\text{ Biofilm inhibition}=\left(1-\;\frac{\left(\;OD\;570\;sample-OD\;570\;negative\;control\right)}{\left(OD\;570\;positive\;control-OD\;570\;negative\;control\right)}\right)\times 100$$

### MTT assay for measuring anticancer activity

The MTT assay was carried out on HCT 116 cells to determine the anticancer activity of NE, WE, CNWE, NN, NW, CNNW, CN, CW, and CCNW (Zengin et al., 2019). HCT 116 cells were cultivated in a medium containing 10% heat-inactivated FBS, 1% penicillin-streptomycin solution (10,000 units of penicillin and 10 mg of streptomycin in 0.9% NaCl), plus 2 mM L-glutamine. The cells were kept at 37°C in a humidified environment with 5% CO_2_. The HCT 116 cells were sub-cultured in 75 cm^2^ cell culture flasks, and the culture medium was replenished every 3 days. To assess the cytotoxic effects of NE, WE, CNWE, NN, NW, CNNW, CN, CW, and CCNW, using the MTT test, several dilutions between 0 and 1,000 μg/mL were evaluated. In 96-well plates, the HCT 116 cells were seeded at a density of 1 × 10^4^ cells per well and incubated until they reached 90%–95% confluency. Each well was treated with 100 μL of medium containing the desired concentrations of NE, WE, CNWE, NN, NW, CNNW, CN, CW, and CCNW, and incubated for 24 h. Then each well received 20 μL of MTT working solution (5 mg/mL), and cells were incubated for another 4 h. Following the incubation period, the culture media was aspirated and 200 μL of DMSO was added to every well. The absorbance was measured with an ELISA reader (Stat Fax 3200, United States) at a wavelength of 570 nm. The equation below was used to calculate the percentage of viable cells:

% cell viability=OD of sample/OD of control×100

A linear regression equation was used to determine the IC_50_ value, which reflects the concentration where the cell viability decreased by 50%. The equation used for this calculation was Y = Mx + C, where Y represents 50 (the desired viability percentage), and the values of M and C were obtained from the viability plot.

### Statistical analysis

The values were reported as mean ± standard deviation (SD), and each experiment in this study was carried out in triplicate. Statistical analysis was performed utilizing SPSS software (version 21, Chicago, IL, United States). The least significant differences (LSD) and One-way ANOVA test were employed to identify the statistically significant differences with *p*-value of 0.05 or less.

## Results

**FIGURE 1:**
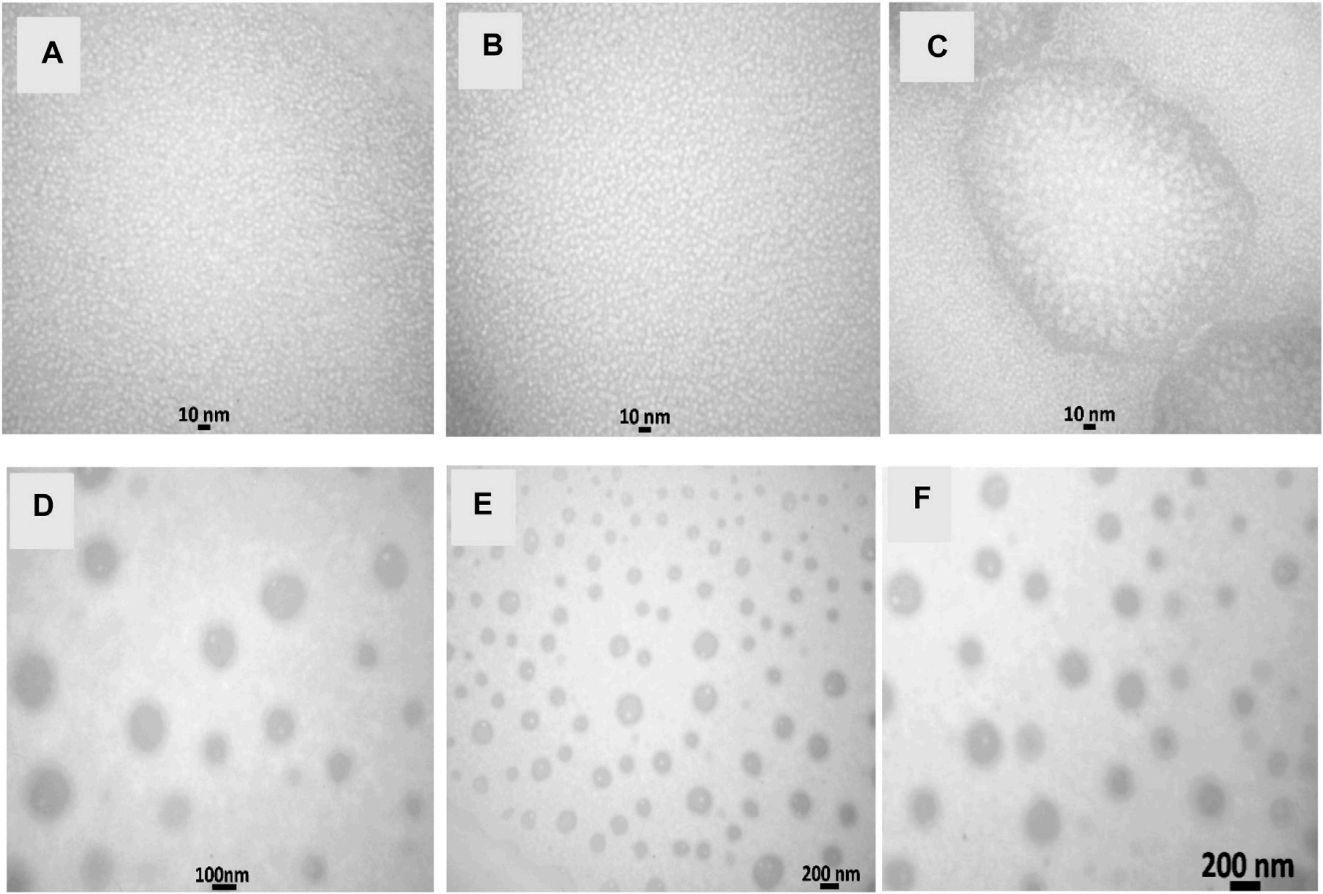
Transmission electron micrographs. **(A)** NN: Nettle nanoemulsion; **(B)** NW: Wormwood nanoemulsion; **(C)** CNNW: Combination of Nettle and Wormwood nanoemulsion; **(D)** CN: nanoencapsulated Nettle; **(E)** CW: nanoencapsulated Wormwood; **(F)** CCNW: combination of nanoencapsulated Nettle and Wormwood.

**FIGURE 2:**
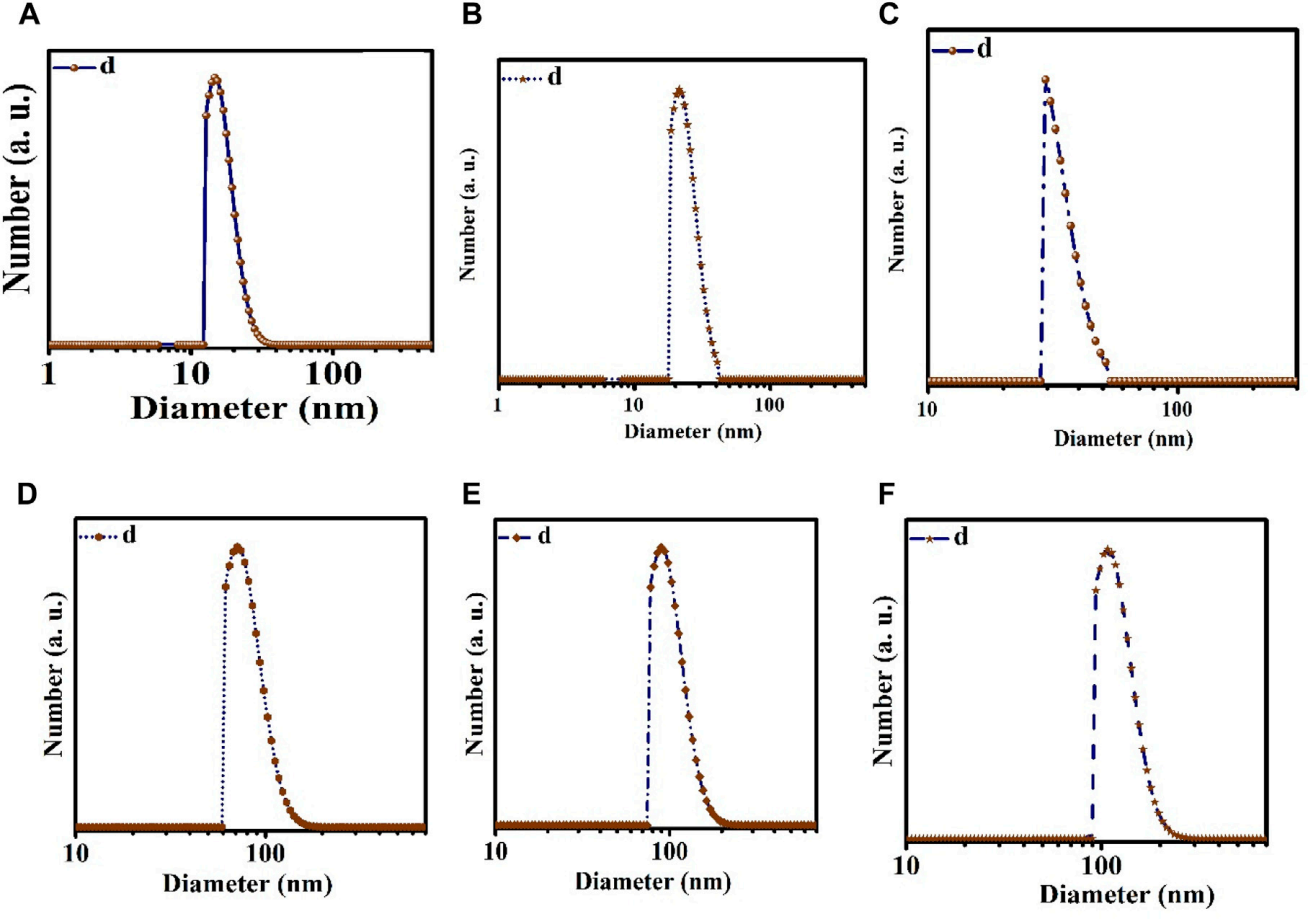
Dynamic light scattering (DLS). **(A)** NN: Nettle nanoemulsion; **(B)** NW: Wormwood nanoemulsion; **(C)** CNNW: Combination of Nettle and Wormwood nanoemulsion; **(D)** CN: nanoencapsulated Nettle; **(E)** CW: nanoencapsulated Wormwood; **(F)** CCNW: combination of nanoencapsulated Nettle and Wormwood.

The TEM and DLS characterization of the prepared nanoemulsion and nanoencapsulation is shown in Figure 1 and Figure 2. The results obtained from DLS analysis, which provided droplet size measurement, were further confirmed by the TEM images. The TEM micrographs (Figure 1) show droplets with diameters ranging from 10 to 30 nm, 20–40 nm, and 30–50 nm for NN (Nettle nanoemulsion), NW (Wormwood nanoemulsion), and CNNW (combination of Nettle and Wormwood nanoemulsion), respectively. The droplet sizes for CN (nanoencapsulated Nettle), CW (nanoencapsulated Wormwood), and CCNW (combination of nanoencapsulated Nettle and Wormwood) were 60–100 nm, 70–110 nm, and 90–110 nm, respectively.

The IZ, MIC, and MBC values for antibacterial activity of NE, WE, CNWE, NN, NW, CNNW, CN, CW, and CCNW are shown in Tables 1, 2. CNNW and CCNW, at high concentrations, showed significantly larger IZ values against the two bacterial strains compared to other treatments (*p* < 0.05). The investigated bacterial strains were resistant to at least some of the pure herbal extracts. At the concentration 380 μg/mL, *S. typhimurium* was found to be more susceptible to CCNW and CNNW compared to *K. pneumoniae* (*p* < 0.05). In addition, CW, CN, NW, CNNW and CCNW, at 380 and 190 μg/mL concentrations, showed significantly larger IZ values against the two tested bacterial strains compared to gentamicin 10 µg as a control group (*p* < 0.05). Overall, the MIC and MBC values of NN were generally lower compared to the other treatments. The studied bacteria had MIC values of all agents between 11.25 and 95 μg/mL along with MBC values between 11.25 and 190 μg/mL. The inhibition percentage of biofilm formation is summarized for NE, WE, CNWE, NN, NW, CNNW, CN, CW, and CCNW against *S. typhimurium* and *K. pneumoniae* in Figure 1. All of the investigated sub-inhibitory concentrations (0.5 MIC) reduced the biofilm formation of both bacteria. However, all the concentrations of CN, CW, and CCNW showed more noticeable effects on *K. pneumoniae* and *S. typhimurium* compared to the other treatments (*p* < 0.05). At a concentration 2 MIC of all agents, *K. pneumoniae* was found to be more susceptible to inhibition of biofilm formation compared to *S. typhimurium* (*p* < 0.05). The maximum inhibitory effects of CCNW were observed on *K. pneumoniae* ( ∼ 78.50%) and *S. typhimurium* ( ∼ 73%). In descending order, the inhibition of biofilm formation was CCNW > CW > CN > CNNW > NN > NW > CNWE > NE > WE against the tested bacteria.

**TABLE 1:** Antibacterial activity using disc diffusion method of hydroethanolic extract, nanoemulsion and nanoencapsulation of Nettle and Wormwood.

Samples	Concentration (µg/mL)	Inhibition zone (mm)	
		*S. typhimurium*	*K. pneumoniae*
NE	380	7 ± 2^Aa^	8.5 ± 1^Aa^
	190	5.5 ± 1^Aa^	6 ± 1^Ab^
	95	4.5 ± 1^Aa^	3.5 ± 1^Ac^
	47.5	2 ± 0^Ab^	2 ± 0^Ad^
WE	380	8 ± 1^Aa^	7.5 ± 1^Aa^
	190	6.5 ± 1^Aa^	5 ± 2^Ab^
	95	4.5 ± 1^Aa^	3.5 ± 1^Ac^
	47.5	3 ± 0^Ac^	2 ± 0^Bd^
CNWE	380	12 ± 1^Ad^	11 ± 1^Ae^
	190	8 ± 1^Aa^	10 ± 2^Aa^
	95	6.5 ± 1^Aa^	7.5 ± 1^Aa^
	47.5	3.5 ± 0^Ac^	5.5 ± 1^Ab^
NN	380	11 ± 2^Aa^	9 ± 1^Aa^
	190	7.5 ± 1^Aa^	7 ± 1^Aa^
	95	4 ± 0^Ae^	5 ± 0^Bb^
	47.5	3 ± 0^Ac^	3.5 ± 0^Ac^
NW	380	14 ± 2^Ad^	13 ± 1^Ae^
	190	12.5 ± 1^Ad^	12 ± 2^Ae^
	95	10 ± 1^Aa^	11 ± 1^Ae^
	47.5	8 ± 1^Aa^	8 ± 1^Aa^
CNNW	380	17 ± 2^Ag^	12 ± 2^Be^
	190	14 ± 2^Ad^	10.5 ± 1^Ba^
	95	10 ± 1^Aa^	8 ± 1^Ba^
	47.5	8.5 ± 1^Aa^	6.5 ± 1^Aa^
CN	380	15 ± 2^Ad^	14 ± 2^Ag^
	190	12.5 ± 1^Ad^	12 ± 1^Ae^
	95	8 ± 1^Aa^	8.5 ± 1^Aa^
	47.5	6 ± 0^Aa^	5.5 ± 1^Ab^
CW	380	15 ± 2^Ad^	14 ± 1^Ag^
	190	13 ± 2^Ad^	12.5 ± 1^Ae^
	95	10 ± 1^Aa^	10.5 ± 1^Ae^
	47.5	8 ± 1^Aa^	9 ± 1^Aa^
CCNW	380	19 ± 1^Ag^	15 ± 2^Bg^
	190	15 ± 1^Ag^	13.5 ± 1^Ag^
	95	11 ± 1^Ad^	10 ± 1^Aa^
	47.5	5 ± 0^Aa^	7 ± 0^Bb^
Gentamicin	10 µg	10 ± 1^Aa^	10.5 ± 1^Aa^

NE, nettle hydroethanolic extract; WE, wormwood hydroethanolic extract; CNWE, combination of nettle and wormwood hydroethanolic extract; NN, nettle nanoemulsion; NW, wormwood nanoemulsion; CNNW, combination of nettle and wormwood nanoemulsion; CN, nanoencapsulated nettle; CW, nanoencapsulated wormwood; CCNW, combination of nanoencapsulated Nettle and Wormwood. The letters a, b, c and etc. denote differences between groups (in column) that are statistically significant at *p* < 0.05. The letters A and B denote differences between groups (in row) that are statistically significant at *p* < 0.05.

**TABLE 2:** Minimum inhibitory concentration (MIC) and minimum bactericidal concentration (MBC) of hydroethanolic extract, nanoemulsions and nanoencapsulations of Nettle and Wormwood against two bacteria.

Samples	*S. typhimurium*		*K. pneumoniae*	
	MIC (µg/mL)	MBC (µg/mL)	MIC (µg/mL)	MBC (µg/mL)
NE	47.5	95	95	190
WE	47.5	95	95	190
CNWE	24.3	48.6	12.1	24.2
NN	11.25	45	11.25	45
NW	30	60	15	30
CNNW	25	50	25	50
CN	35	70	17.5	35
CW	22.5	45	35	70
CCNW	45	90	22.5	45

NE, nettle hydroethanolic extract; WE, wormwood hydroethanolic extract; CNWE, combination of nettle and wormwood hydroethanolic extract; NN, nettle nanoemulsion; NW, wormwood nanoemulsion; CNNW, combination of nettle and wormwood nanoemulsion; CN, nanoencapsulated Nettle; CW, nanoencapsulated Wormwood; CCNW, combination of nanoencapsulated Nettle and Wormwood.

**FIGURE 3:**
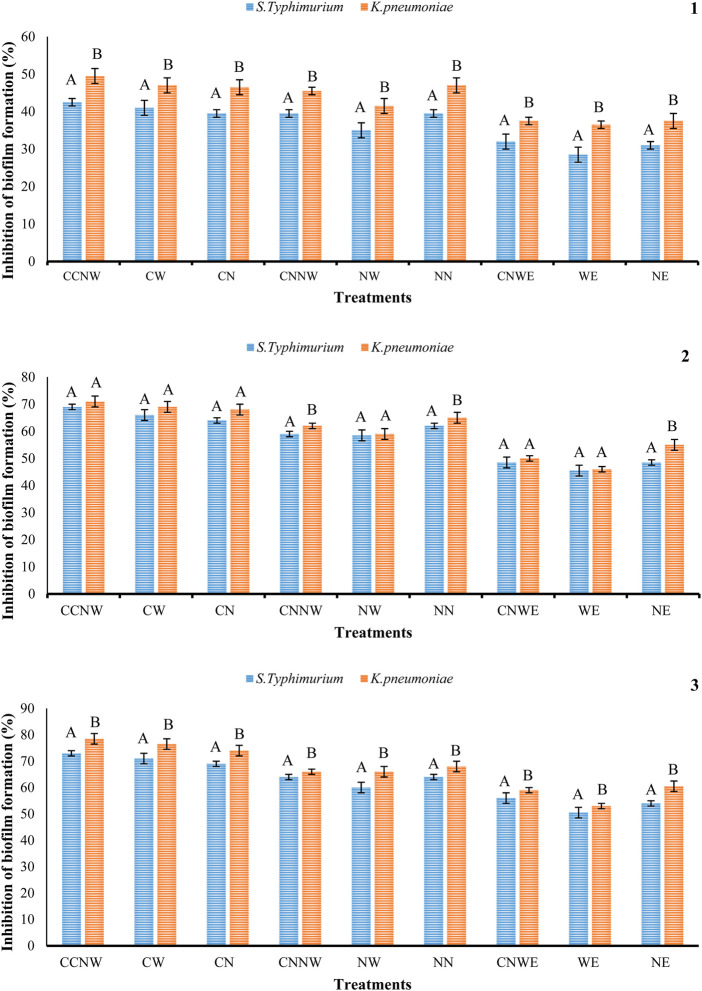
Percentage inhibition of *S. typhimurium*, and *K. pneumonia* biofilm formation with the various treatments. NE: Nettle hydroethanolic extract, WE: Wormwood hydroethanolic extract, CNWE: Combination of Nettle and Wormwood hydroethanolic extract, NN: Nettle nanoemulsion, NW: Wormwood nanoemulsion, CNNW: Combination of Nettle and Wormwood nanoemulsion, CN: nanoencapsulated Nettle, CW: nanoencapsulated Wormwood, CCNW: combination of nanoencapsulated Nettle and Wormwood. The letters A and B denote differences between groups that are statistically significant at *p* < 0.05. 1: biofilm formation at ½ MIC concentration, 2: biofilm formation at MIC concentration, 3: biofilm formation at 2 MIC concentration.

**FIGURE 4:**
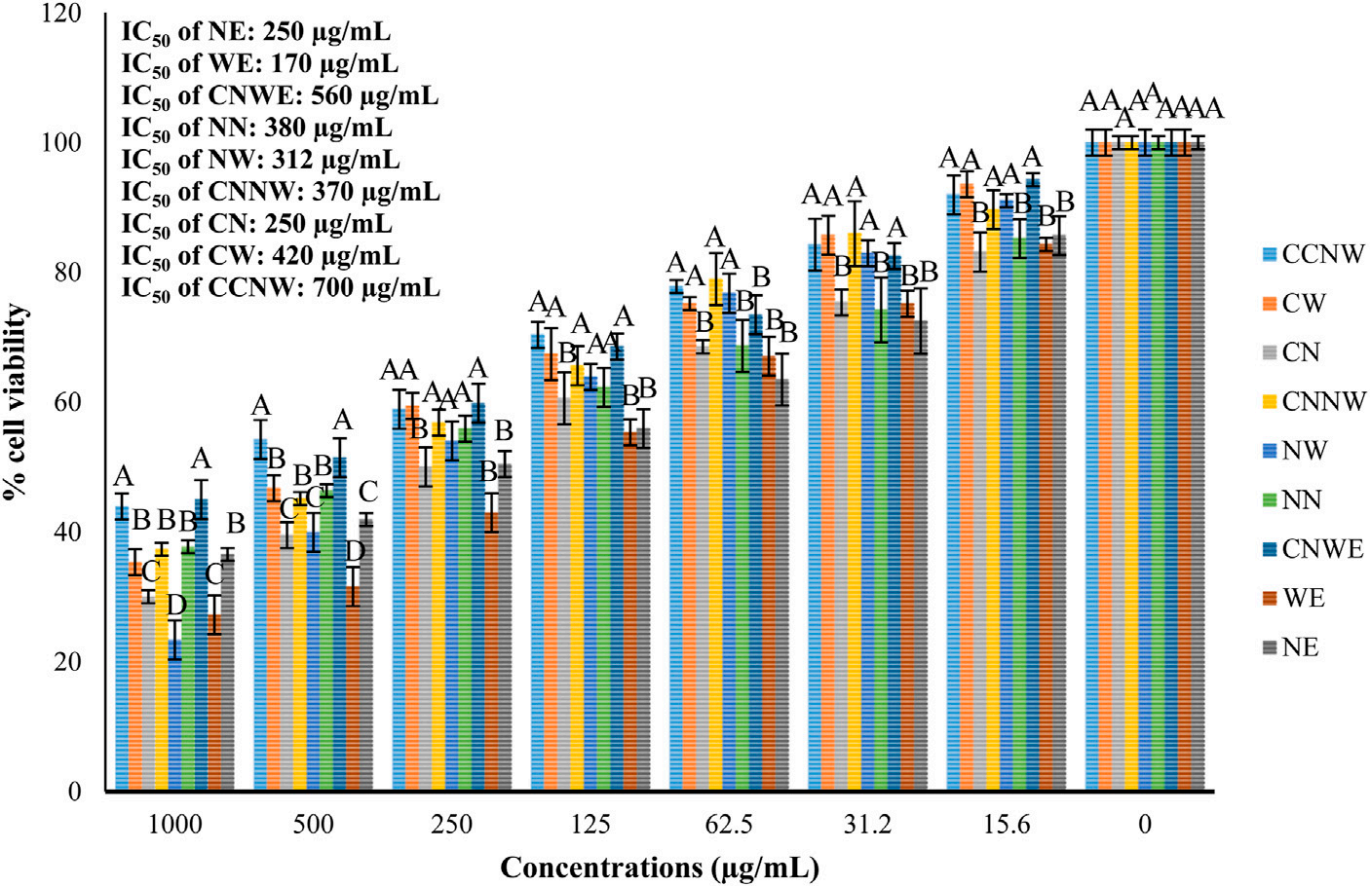
Effect of various treatments on the viability of colon cancer cell line (HCT 116). NE: Nettle hydroethanolic extract, WE: Wormwood hydroethanolic extract, CNWE: Combination of Nettle and Wormwood hydroethanolic extract, NN: Nettle nanoemulsion, NW: Wormwood nanoemulsion, CNNW: Combination of Nettle and Wormwood nanoemulsion, CN: nanoencapsulated Nettle, CW: nanoencapsulated Wormwood, CCNW: combination of nanoencapsulated Nettle and Wormwood. The letters A and B denote differences between groups that are statistically significant at *p* < 0.05.

Figure 3 shows the results of cell viability of HCT 116 cells following a 24-h exposure to the tested agents. The findings show that all the agents, including NE, WE, CNWE, NN, NW, CNNW, CN, CW, and CCNW, resulted in a substantial decrease in cell viability within the 24-h timeframe. The IC_50_ values of NE, WE, CNWE, NN, NW, CNNW, CN, CW, and CCNW were determined to be 250, 170, 560, 380, 312, 370, 250, 420, and 700 μg/mL, respectively. Cell viability assays indicated that exposure to a high concentration of NW resulted in a significantly lower cell viability compared to other groups (*p* < 0.05). However, as compared to other groups, the most pronounced drop in cell viability was seen with 500 μg/mL concentration of WE (*p* < 0.05). Figure 4 shows a dose dependent cytotoxic effect with all concentration of agents against HCT 116 (*p* < 0.05).

## Discussion

In recent years, plant extracts have been investigated as additives in the food industry, as well as for therapeutic applications due to their rich content of bioactive compounds, i.e., polyphenols and carotenoids that possess antioxidant and antimicrobial activity (Chaleshtori et al., 2015; Proestos, 2020). The secondary metabolites of different plants, including essential oils, extracts, and their nanoemulsion and nanoencapsulation forms, have been widely investigated for their biological actions. As a result, they have undergone extensive testing and are now used in many different applications, including pharmacology, medical sciences, pharmaceutical botany, clinical microbiology, food preservation and phytopathology (Dinda et al., 2016; Sharafati Chaleshtori and Sharafati Chaleshtori, 2017).

Earlier research reported that Nettle-mediated biosynthesis of silver nanoparticles (AgNPs) showed potent antibacterial activity against pathogenic microorganisms. Furthermore, the anticancer effect of the AgNPs was assessed by determining the IC_50_ dose using the XTT assay against MCF-7 breast cancer cells with an identified IC_50_ value of 0.243 ± 0.014 μg/mL (Dağlıoğlu et al., 2023b). Hashem and Salem (2022) reported that biosynthesized selenium nanoparticles (SeNPs) from an aqueous extract of *U. dioica* leaves indicated potent antibacterial activity against both Gram-negative and Gram-positive bacteria, along with multi-cellular and unicellular fungi. The MIC of the SeNPs against *E. coli*, *Pseudomonas aeruginosa*, *B. subtilis*, as well as *Staphylococcus aureus* were 250, 31.25, and 500 μg/mL, respectively, whilst the MIC values against *C. albicans*, *A. fumigatus*, *A. niger*, and *A. flavus* were 62.5, 15.62, 31.25, and 7.81 μg/mL, respectively. Moreover, the cytotoxicity of SeNPs on the normal Vero cell line CCL-81, showed an IC_50_ value of 173.2 μg/mL (Hashem and Salem, 2022). Our results showed that NE, NN, and CN exhibited antibacterial activity against *S. typhimurium* and *K. pneumoniae*. The MIC and MBC values for these agents ranged from 11.25 to 95 μg/mL. Additionally, the cytotoxicity of NE, NN, and CN on HCT 116 cells using the MTT assay showed IC_50_ values of 250, 380, and 250 μg/mL, respectively.

Evaluating the combined impact of Nettle and Wormwood extracts on pathogenic bacteria was a crucial focus of this study. Previous studies have demonstrated that the simultaneous utilization of various herbal extracts has a stronger antimicrobial effect than the use of a single extract. Zarei Yazdeli et al. (2021), demonstrated the potent combination of methanolic extracts from *Dracocephalum kotschyi* and *Trachyspermum ammi* in effectively inhibiting the growth of *P*. *aeruginosa*, *Shigella dysenteriae*, *E*. *coli*, and *S*. *aureus* through a synergistic antimicrobial effect (Zarei Yazdeli et al., 2021). Our results, specifically for CNWE, align with previous research. When multiple antimicrobial agents are used together, they can have a synergistic, antagonistic, or indifferent effect on microbial populations compared to their individual effects. One theory to explain the synergy is that one substance aids in binding another substance to its target site by altering the structure of the cell (Acharjee et al., 2023).

Many hundreds of plant polyphenols have been isolated with diverse molecular structures, typically featuring an aromatic ring with one or multiple hydroxyl groups plus other substituents. These compounds possess beneficial properties like antimicrobial, antioxidant, anticancer, and anti-inflammatory effects (Leri et al., 2020). Plant polyphenols could act as antimicrobial agents against antibiotic-resistant pathogens. These compounds can negatively interfere with various structural and biochemical attributes of microbes, disrupting the normal functioning of cell membranes, cytoplasmic contents, proteins as well as enzymes. As a result, the microbial metabolism is disturbed, leading to failure to grow or to outright cell death (Prakash et al., 2018).

Nanoparticles possess distinct physicochemical characteristics, including high reactivity, a huge surface area to mass ratio, ultrasmall and controlled size, along with a structure that can be functionalized (Onyeaka et al., 2022). Some nanomaterials are used as carrier agents, such as chitosan, cellulose, dextran, starch, cyclodextrin and alginate, but these also possess antimicrobial activity. They achieve this by disrupting membrane potential, generating reactive oxygen species, along with altering metabolic reactions (Kujur et al., 2017; Chen et al., 2023). Nanoemulsions, due to their tiny size and large surface area per unit volume, allow efficient transport through porin proteins in the outer membrane. This enables the effective delivery of herbal components (essential oils or extracts) because the hydrophilic groups of the emulsified molecules are exposed during the process (Barzegar et al., 2023). Furthermore, nanoencapsulation offers a way to control stability, solubility, bioavailability, and promote the release of bioactive components. Numerous encapsulation techniques for natural bioactive components have been demonstrated to be efficient methods to enhance their absorption both *in vitro* and *in vivo* (Mukurumbira et al., 2022; Reis et al., 2022).

One study demonstrated that the combined hydroethanolic extracts of *A. absinthium* and *C. paradisi* exhibited antibacterial activity against *E. coli* with a zone of inhibition of 5.6 ± 0.06 mm. However, this treatment did not show any antibacterial activity against *S. aureus*, *K. pneumoniae*, or *S. enterica*. The MTT findings showed that while neither plant extract had an affected the Vero cell line, they had a cytotoxic effect on the Huh-7 cell line (Ali et al., 2021). Sofi et al. (2022) reported that *A. absinthium* leaf methanolic extract displayed the highest activity against *E. faecalis* (20 ± 0.7 mm) as well as *E. coli* (18 ± 0.8 mm), followed by *P. aeruginosa* (16 ± 0.6 mm), *C. albicans* (14 ± 0.9 mm), and *S. aureus* (13 ± 0.8 mm), with MIC values of 128, 128, 128, 256, and 256 μg/mL, respectively. Moreover, this extract exhibited substantial (*p* ≤ 0.05) cytotoxicity against the A549 cancer cell line (human lung cancer) with an IC_50_ value of 36.8 μg/mL (Sofi et al., 2022). In agreement with above studies, we observed a significant antibacterial activity against *S. typhimurium* and *K. pneumonia* especially for the nanoencapsulation form of WE, Moreover anticancer activity again HCT 116 was observed for the nanoemulsion form of WE (cell viability = 23.37% for 1,000 μg/mL concentration; *p* < 0.05). A previous study showed that the leaf and stem ethanolic extracts of *A. absinthium* and *A. annua* showed significant antibacterial activity against carbapenem-resistant *Klebsiella* spp., extended-spectrum β-lactamases *E. coli* (ESBL) and ESBL *Klebsiella* spp. (Bordean et al., 2023). In our study, the highest antibacterial activity (MIC = 47.5 μg/mL, MBC = 95 μg/mL, IZ = 8 ± 1 mm and 50.5% inhibition of biofilm at 2 MIC concentration) was recorded for WE against *S. typhimurium*. However, we found that the nanoemulsion, and encapsulation forms of WE had significantly higher antibacterial and anticancer activity than WE alone (*p* < 0.05). Overall, the antibacterial activity of CCNW against the two tested bacteria was higher than the other agents.

Several compounds, including lommen, vanillic acid, chlorogenic acid and epichatechin have been found in various species of the genus *Asteraceae*. Recent studies have shown that chlorogenic acid attacks the outer membrane of bacteria, disrupts it, depletes the intracellular potential, and releases macromolecules from the cytoplasm, all leading to cell death (Mamatova et al., 2019). Vanillic acid was found to cause changes in the intracellular ATP concentration, intracellular pH, and membrane potential (Qian et al., 2020). Flavonoids have several hydroxyl groups and show a pronounced tendency to bind to proteins. The inhibition of the binding of KpDnaB (*K. pneumoniae* DnaB helicase) to dNTPs (deoxyribonucleoside triphosphates) by flavonols in *K. pneumoniae,* could possibly explain their antibacterial activity (Chen and Huang, 2011).

It is possible that all the extracts, nanoemulsion, and encapsulation forms led to the accumulation of lipid peroxidase (LPO) products accompanied by a decrease in intracellular enzymatic and non-enzymatic antioxidants, resulting in increased LPO-induced apoptosis. Moreover, these agents may induce mitochondrial changes in HCT 116 cells, leading to the translocation of cytochrome C to the cytosol along with caspase activation, which would subsequently trigger apoptosis (Thakur and Devaraj, 2020). Our findings are consistent with some of the previously mentioned studies, even though there are differences in bacterial strains, cancer cell lines, testing methods, and the use of nanoemulsion and encapsulation forms of the hydroethanolic extracts.

## Conclusion

In conclusion, our finding confirmed that the combined extracts from both plants, as well as the combined extract in nanoemulsion and nanoencapsulation forms, showed the most pronounced antibacterial activity against *K. pneumoniae* and *S. typhimurium,* as well as the highest anticancer activitiy against HCT 116 colon cancer cells. The limitations of the study were that the cell viability assays for bacteria and HCT 116 cells were not sufficiently sensitive and the time points evaluated were insufficient to assess alterations in biofilm growth for bacteria, and subtle pH variations may have affected the results. Further validation of these results will be required using other methods, such as monitoring gene expression in cell lines (biofilm-related genes and colon cancer genes) and eventually carrying out studies in animal models.

## Data Availability

The raw data supporting the conclusion of this article will be made available by the authors, without undue reservation.
